# Self-concept and body image dissatisfaction in West Australian adolescent boys and girls

**DOI:** 10.1186/2050-2974-2-S1-O58

**Published:** 2014-11-24

**Authors:** Katerina Chin-A-Loy, Monique Robinson, Karina Allen, Peter Jacoby, Neil McLean

**Affiliations:** 1 School of Psychology, The University of Western Australia, Perth, Australia; 2 Telethon Kids Institute, Perth, Australia

## 

Discrepancies between current body image perception and desired body image represent levels of body image dissatisfaction (BID). This study investigated the relationship between BID and multidimensional self-concept in adolescents from the Western Australian Pregnancy Cohort (Raine) Study. We hypothesized that BID is influenced by specific domains of the self-concept with gender differences. The Raine Study 14-year follow-up involved 1425 Caucasian adolescents (744 boys, 681 girls). The Body Figure Perception Scale explored adolescents' body image. Harter's Self-Perception Profile for Adolescents measured self-concept through 9 domains. Data analysis was performed using linear regression models. We found that 38% of boys wanted to be thinner, and 21% wanted to be bigger; while 63% of girls wanted to be thinner, and 6% wanted to be bigger. For both genders, the Physical Appearance domain was strongly associated with BID. In boys, low Athletic Competence was related to higher levels of BID. In girls, low Global Self-worth, Athletic Competence, and Close Friendship domains were associated with higher levels of BID. The relationship Athletic Competence-BID was attenuated, in both genders, when models were adjusted for body mass index. How adolescents view themselves in terms of their self-concept has an impact on BID with differences by gender. These findings could inform prevention and intervention initiatives in the field.

This abstract was presented in the **Disordered Eating and Body Image** stream of the 2014 ANZAED Conference.

